# MicroRNAs modulation by isodrimeninol from *Drimys winteri* in periodontitis-associated cellular models: preliminary results

**DOI:** 10.3389/froh.2025.1489823

**Published:** 2025-05-21

**Authors:** Nelia M. Rodríguez, Pía Loren, Isis Paez, Viviana Burgos, Constanza Martínez-Cardozo, Alejandra Chaparro, Luis A. Salazar

**Affiliations:** ^1^ Doctoral Program in Sciences, Major in Applied Cellular and Molecular Biology, Universidad de La Frontera, Temuco, Chile; ^2^ Center of Molecular Biology and Pharmacogenetics, Department of Basic Sciences, Faculty of Medicine, Temuco, Chile; ^3^ Department of Veterinary Sciences and Public Health, Faculty of Natural Resources, Universidad Católica de Temuco, Temuco, Chile; ^4^ Department of Biological and Chemical Sciences, Faculty of Natural Resources, Universidad Católica de Temuco, Temuco, Chile; ^5^ Department of Biomedical Sciences, Ethics, Research and Education, Faculty of Dentistry, Universidad de los Andes, Santiago, Chile; ^6^ Center for Biomedical Research and Innovation (CIIB), Universidad de los Andes, Santiago, Chile; ^7^ Department of Oral Pathology and Conservative Dentistry, Periodontics, Faculty of Dentistry, Universidad de los Andes, Santiago, Chile

**Keywords:** microRNAs, inflammation, periodontitis, isodrimeninol, citokines

## Abstract

**Introduction:**

Periodontitis is a chronic inflammatory disease characterized by the progressive destruction of the tooth's supporting tissues, driven by complex interactions between periodontopathogenic bacteria, environmental factors, and the host immune response. MicroRNAs (miRNAs) have emerged as key modulators of inflammatory pathways and are increasingly recognized for their role in the pathogenesis of periodontitis. Their deregulation in this disease suggests potential therapeutic applications targeting miRNA expression. Natural compounds such as isodrimeninol, derived from *Drimys winteri* (*Dw*), may offer novel approaches to modulate miRNA activity due to their antiinflammatory properties. However, no studies have previously linked this sesquiterpene to miRNA regulation in periodontitis. This study investigates the *in vitro* effects of isodrimeninol on six miRNAs (miR-17-3p, miR-21-3p, miR-21-5p, miR-146a-5p, miR-155-5p, and miR-223-3p) associated with periodontitis using two cellular models.

**Methods:**

Saos-2 cells (osteoblast-like cells) and periodontal ligament-derived mesenchymal stromal cells (hPDL-MSCs). Both cell types were stimulated with lipopolysaccharide (LPS) to induce inflammation and treated with isodrimeninol and resveratrol for comparison.

**Results:**

Isodrimeninol reduced Interleukin-1beta (IL-1β) and Interleukin-6 (IL-6) gene expression and caused differential expression patterns of the miRNAs examined, upregulating miR-146a-5p and miR-223-3p, while downregulating miR-17-3p, miR-21-3p, miR-21-5p, and miR-155-5p (*p* < 0.05).

**Conclusion:**

These findings indicate a connection between miRNAs, periodontitis, and the regulation of inflammation by isodrimeninol, providing potential opportunities for the treatment. However, further validation is needed to confirm these results.

## 1 Introduction

Periodontitis is the most common chronic inflammatory disease in humans. It is a major public health issue causing tooth loss, disability, masticatory dysfunction, poor nutritional status, reduced quality of life, and serious economic problems (1). According to a 2015 disease burden study, periodontitis is the sixth most prevalent disease worldwide (2), and this prevalence rate has shown an increasing trend in recent years (3). Periodontitis is a multifactorial disease caused by complex interactions between specific bacterial pathogens, destructive host immune responses, and environmental factors (4, 5) that lead to chronic inflammation of the periodontal tissues (6). It has been concluded that the inflammatory response of the host against bacteria and their virulence factors, especially lipopolysaccharide (LPS) (7), is the basis for understanding the pathogenesis of periodontitis since the body's destructive response is related to an elevated expression of inflammatory cytokines in the tissue.

MicroRNAs (miRNAs) are small, non-coding, single-stranded RNA molecules ranging from 18 to 22 nucleotides in length. They regulate gene expression at the post-transcriptional level by either inhibiting the translation or promoting the degradation of their target mRNAs (8). Several miRNAs are potentially linked to inflammatory processes in periodontitis and warrant detailed exploration. For instance, miR-146a and miR-21 have been implicated in regulating inflammation by modulating cytokine production and NF-κB signaling pathways, which are pivotal in periodontal disease progression (9, 10). Their dysregulation by oral bacterial plaque components contributes to periodontitis pathogenesis, affecting both innate and adaptive immune responses (11, 12). Significant miRNA level changes are observed in diseased vs. healthy tissues, offering diagnostic and prognostic potential for periodontal disease (13–15). For instance, miR-155 and miR-223 are overexpressed in neutrophils from periodontally diseased tissues, modulating cell adhesion and chemokine mRNA stability, thereby influencing neutrophil migration and function (16). Additionally, miR-21, which is elevated in patients with periodontitis, exhibits anti-inflammatory properties by inhibiting the production of pro-inflammatory cytokines in macrophages stimulated by *Porphyromonas gingivalis* lipopolysaccharides. Its deficiency is associated with increased gingival inflammation and alveolar bone loss in murine models (9). Despite promising therapeutic prospects, further research is needed to elucidate miRNA-mediated inflammation regulation in periodontitis.

Current treatments often view periodontal diseases as opportunistic infections influenced by host inflammatory responses. However, conventional therapies face challenges like antimicrobial resistance (17). In this regard, there is evidence that nutraceuticals and medicinal compounds isolated from plants are beneficial to health by preventing and treating diseases (18, 19). In this study, we focused our attention on isodrimeninol, a sesquiterpene with a drimane skeleton isolated from bark extracts of *Drimys winteri* or Canelo, a tree native to south-central Chile and Argentina considered sacred by the Mapuche people (20). Isodrimeninol has been shown to have an anti-inflammatory effect on atherosclerosis (21, 22). Although not directly linked to periodontitis, its potential anti-inflammatory effects suggest it could modulate periodontal disease processes. We compared its anti-inflammatory effect with resveratrol, a phytoalexin compound found in plants that has been shown to reduce inflammation in human gingival tissue, promoting the proliferation and osteogenic differentiation capacity of human gingival mesenchymal stromal cells, improving their immunomodulation, and demonstrating a positive effect in the treatment of periodontitis by inhibiting the infiltration of inflammatory cells in human inflamed gingival tissues (23). Thus, *Dw* and isodrimeninol could serve as natural therapeutic alternatives for the treatment of periodontitis by regulating the expression of inflammatory molecules and microRNAs that are dysregulated in this disease.

As a research team, we pose the following question: How does isodrimeninol modulate microRNA expression and inflammatory processes in periodontitis, and what are the implications for developing novel therapeutic strategies? The primary objective of this study is to evaluate the *in vitro* effects of isodrimeninol on the expression of specific microRNAs associated with periodontitis, utilizing Saos-2 and human periodontal ligament mesenchymal stromal cells (hPDL-MSCs) as cellular models. Saos-2 cells, an osteoblastic cell line renowned for its high biomineralization capacity and ability to deposit a mineralization-competent extracellular matrix, serve as an ideal model for studying osteoblastic differentiation (24). Additionally, hPDL-MSCs were selected due to their pivotal role in periodontal tissue regeneration, including their capacity to differentiate into osteoblasts and contribute to bone healing (25). The justification for this investigation stems from the paucity of studies examining the anti-inflammatory effects of isodrimeninol in the context of periodontitis, despite its potential as a natural therapeutic agent. Notably, this study offers a novel perspective by exploring microRNA expression as a potential mechanism through which isodrimeninol exerts its effects, thereby providing new insights into its potential roles in disease-associated processes and the development of targeted therapies.

## 2 Materials and methods

### 2.1 Selection of pro-inflammatory cytokines and miRNAs associated with periodontitis

Based on an updated search of the Scopus, PubMed, and Medline databases, six miRNAs were identified as deregulated in periodontitis. These miRNAs are related to inflammatory genes such as TNF-α, Il-6, and IL-1β. They are involved in the regulation of the nuclear factor-κB (NF-κB), nuclear factor erythroid 2 (Nrf2), and mitogen-activated protein kinase (MAPK) signaling pathways. They are presented in Table 1.

**Table 1:** MiRNAs deregulated in periodontitis.

Selected miRNA	Expression	Reference
hsa-miR-21-3p	Increased	(9)
hsa-miR-21-5p	Increased	(9)
hsa-miR-146a-5p	Increased	(26)
hsa-miR-155-5p	Decreased	(14)
hsa-miR-223-3p	Increased	(16)
hsa-miR-17-3p	Increased	(27)

### 2.2 Pro-inflammatory cytokine expression in saos-2 cells and periodontal ligament-derived mesenchymal stromal cells

#### 2.2.1 Cell culture

The Saos-2 cell line and hPDL-MSCs were used as periodontitis study models, which were kindly donated by Dr. Constanza Martínez from the Universidad de Los Andes, Santiago, Chile, who previously characterized periodontal ligament-derived mesenchymal stromal cells by flow cytometry and differentiation potential (28). Saos-2 cells correspond to a primary human osteosarcoma cell line used as osteoblasts (29), and hPDL-MSCs was obtained by periodontal ligament explants from the middle third root of third molars of a 27-year-old man. Both cell types were cultured in Dulbecco's Modified Eagle's Medium (DMEM) supplemented with 10% fetal bovine serum (FBS) and a mix of antibiotics (100 U/ml penicillin, 100 µg/ml streptomycin) and antifungals (0.25 µg/ml amphotericin B). HPDL-MSCs were used in passages 2–5. Both cell cultures were maintained at 37°C in a humidified atmosphere containing 5% CO_2_. The culture medium was replaced twice a week, and trypsinization was performed when the cells reached 70%–80% confluent cultures.

#### 2.2.2 Effect of treatment with different doses of *Drimys winteri* isodrimeninol and resveratrol on the expression of pro-inflammatory cytokines and miRNAs in saos-2 cells and hPDL-MSCs

Inflammatory modeling for both Saos-2 cells and hPDL-MSCs was performed as follows: Both cell types were inflamed with 1 µg/ml LPS for 24 h and subsequently incubated at isodrimeninol concentrations (6.25 and 12.5 µg/ml) and resveratrol concentrations (5.71 and 11.41 µg/ml) for 24 h. In addition, LPS-stimulated control cells not exposed to the treatments and LPS-stimulated cells administered only to the treatment vehicle were also used. Isodrimeninol and resveratrol were dissolved in DMSO. These treatments were carried out to evaluate gene expression of the IL-6, IL-1β, and TNF-α genes.

### 2.3 Molecular analysis

#### 2.3.1 Total RNA extraction

Saos-2 cells and hPDL-MSCs were seeded in 24-well plates at a concentration of 1 × 10^5^ cells/well. Subsequently, they were treated with the above-mentioned conditions (50 ng and 1 μg/ml of LPS for 24 h). The gene expression of the inflammatory cytokines IL-6, TNF-α, and IL-1β was assessed to test the inflammatory process. The culture medium was removed after producing a cell suspension containing 1 × 10^5^ cells/ml with the previously described treatments. Following the manufacturer's instructions, the RNA was isolated using a Trizol reagent (Invitrogen, USA). Concentration was determined by spectrophotometry in an Infinite® M200 PRO NanoQuant Tecan microplate reader (Thermo Fisher Scientific, USA), and purity was determined by measuring absorbance at 260/280 nm, with ratios close to 2 being considered optimal.

#### 2.3.2 MicroRNA extraction

Saos-2 cells and hPDL-MSCs were seeded in 24-well plates at a concentration of 1 × 10^5^ cells/ml. Subsequently, they were treated with 1ug/ml LPS for 24 h, and the inflamed cells were subsequently treated with different concentrations of isodrimeninol (6.25, 12.5, 25, 50 μg/ml) and resveratrol (5.71 and 11.41 µg/ml) as positive control and kept in culture for 24 h. The mirVana^TM^ commercial kit (Invitrogen, Life Technologies) was used according to the manufacturer's specifications, using the protocol for total RNA extraction. Total RNA enriched with small RNAs was quantified on a Nanoquant microplate reader (Thermo Fisher Scientific, USA). Once the miRNAs were extracted, they were stored at −20°C until analysis.

#### 2.3.3 Synthesis of cDNA from total RNA

The cDNA was synthesized from 1 µg of extracted total RNA. According to the manufacturer's instructions, the reverse transcription reaction was carried out using the High-Capacity RNA-to-cDNA™ kit (Applied Biosystems, Foster City, CA, USA). The synthesized cDNA was stored at −20°C until use.

#### 2.3.4 Synthesis of cDNA from extracted miRNAs

From 2 ng of extracted enriched RNA, cDNA synthesis was performed by RT-qPCR, using the TaqMan™ MicroRNA Reverse Transcription Kit (Applied Biosystems, Foster City, CA, USA). After obtaining the synthesized cDNA, it was stored at −20°C until analysis.

#### 2.3.5 Analysis of pro-inflammatory cytokine expression by qRT-PCR

To evaluate the degree of inflammation of Saos-2 cells and hPDL-MSCs stimulated with LPS and subsequently of Saos-2 cells and hPDL-MSCs treated with different doses of resveratrol and isodrimeninol after being stimulated with LPS, differential expression of the TNF-α, IL1-β, and IL-6 genes was determined by q-PCR using Fast® SYBR Green Master Mix (Applied Biosystems). The cDNA obtained in cDNA synthesis from total RNA and primer sequences from Table 2 were used. RPL27 was used as an endogenous reference gene for the study genes. Primers were used at a 200 nM concentration according to previous standardizations (22). The reactions were subjected to the following thermocycling scheme in the StepOne Real-Time PCR System (Applied Biosystems, USA): initial activation at 95°C for 20 s, followed by 40 cycles made up of denaturation cycles at 95°C for 3 s and an annealing/extension step at 60°C for 30 s. Data analysis was performed using the comparative Ct method, obtaining the cycle threshold (Ct) value and subsequent comparison of the Ct between the amount of gene transcript of the samples and the normalizing gene (2-^ΔΔCt^ method). Negative controls were used, and technical and biological triplicates were performed.

**Table 2:** Primer sequences used for the qRT-PCR analysis.

Gene	Accession number	Sequence forward	Sequence reverse	Reference
TNF-α	NM_000594.4	GGCAGGTTCTGTCCCTTTCA	GTCGCGGATCATGCTTTCTG	(21)
IL1-β	NM_000576.3	TGAAGCTGATGGCCCTAAACA	GTGGTGGTCGGAGATTCGTA	(21)
IL-6	NM_000600.5	GAGAGTAGTGAGGAACAAGCCA	GGTCAGGGGTGGTTATTGCAT	(21)
RPL27	NM_000988.5	TCCGGACGCAAAGCTGTCATC	GGTCAATTCCAGCCACCAGAGCAT	(30)

TNF-α, tumoral necrosis factor α; IL1-β, interleukin 1β; IL-6, interleukin 6; RPL27, ribosomal protein L27.

#### 2.3.6 Analysis of miRNA expression by real-time PCR

The TaqMan® miRNA assay system (Life Technologies, CA, USA) was used to quantify miRNA expression in Saos-2 cells and hPDL-MSCs. The real-time PCR reaction was performed under the following conditions: 10 μl of TaqMan Fast Advanced Master Mix, 1 μl of TaqMan Advanced miRNA Assay (Thermo Fisher Scientific) to quantify expression of hsa-miR-17-3p, hsa-miR-21-3p, hsa-miR-21-5p, hsa-miR-155-5p, hsa-miR-223-3p, and hsa-miR-146a-5p (Assay ID.: 477932_mir, 477973_mir, 477975_mir, 483064_mir, 477983_mir and 478399_mir, respectively), 5 μl of diluted cDNA (1:10) and 4 μl of sterile distilled water for a final volume of 20 μl. The relative expression analysis was performed using the comparative ΔΔCt method. As reported in the literature, the hsa-miR-191-5p was used as an endogenous control to normalize the samples (31). The thermocycling protocol consisted of two initial cycles at 50°C for 2 min and 95°C for 10 min. Then, 40 cycles were run at 95°C for 15 s and 60°C for 1 min. The assays were performed in technical and biological triplicate. All assays were performed on a StepOne System (Applied Biosystems, USA) using the StepOne v. 2.2 software.

### 2.4 Statistical analysis

A one-way ANOVA and Dunnett's multiple comparison post-test were used to examine the effect of LPS treatment and, subsequently, LPS-treated cells at different doses of resveratrol and isodrimeninol. Prior to conducting parametric tests, the normality of the data distribution was assessed using the Shapiro–Wilk test for smaller sample sizes (<50). This test verified whether the data conformed to a Gaussian distribution by analyzing deviations from normality based on *p*-values, with significance set at *p* < 0.05. The gene expression data obtained were processed with Excel and then analyzed with the GraphPad Prism version 5.0 statistical program (GraphPad Software Inc., San Diego, CA, USA). All statistical tests of the hypotheses were two-tailed. The significance level was *p* < 0.05

## 3 Results

### 3.1 Effect of isodrimeninol from *Drimys winteri* and resveratrol on the viability of Saos-2 cells and hPDL-MSCs

**Figure 1:**
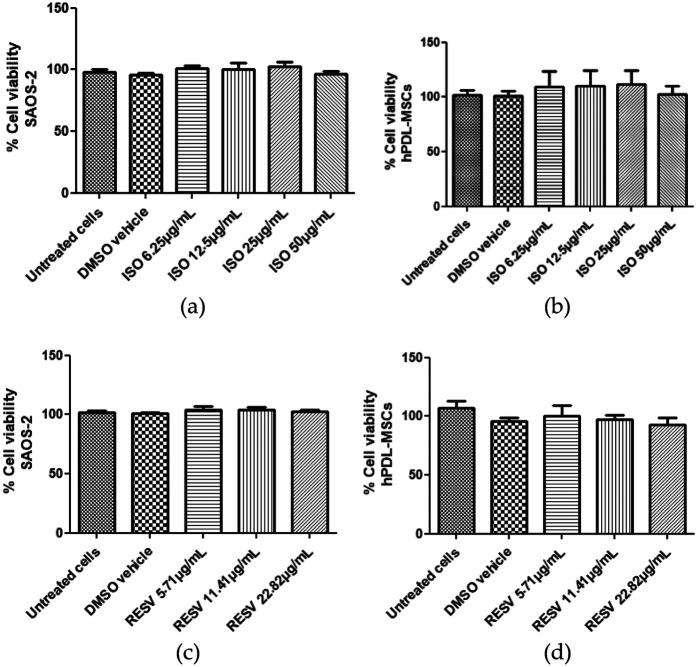
Effect of isodrimeninol from *Drimys winteri* and resveratrol on SAOS-2 cell and hPDL-MSCs viability. Cell viability was developed by MTS assay. Effect of Dw isodrimeninol on the viability of SAOS-2 cells **(a)** and hPDL-MSCs **(b)**. Effect of resveratrol on the viability of SAOS-2 cells **(c)** and hPDL-MSCs **(d)**. Data are expressed as mean ± standard deviation (*n* = 9). Statistical analysis was performed using ANOVA and Dunnett's multiple comparison post-test. CT, control, untreated cells, VH, DMSO vehicle; Iso, isodrimeninol; Resv, resveratrol; LPS, lipopolysaccharide.

**Figure 2:**
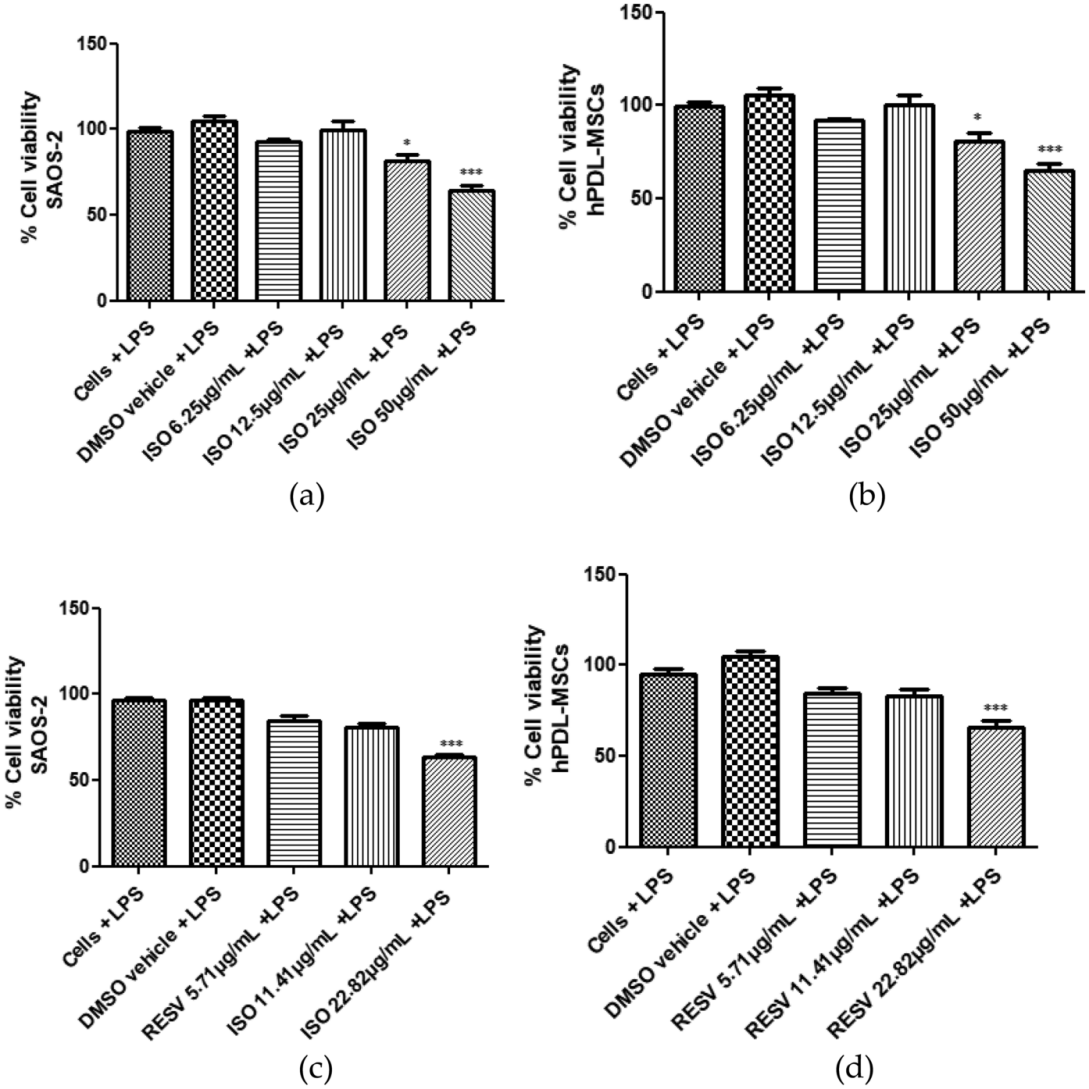
Effect of isodrimeninol from *Drimys winteri* and resveratrol on LPS-stimulated SAOS-2 cell and hPDL-MSCs viability. Cell viability was developed by MTS assay. Effect of isodrimeninol on the viability of SAOS-2 cells stimulated with LPS (1 µg/ml) for 24 h. **(a)** Effect of Dw isodrimeninol on the viability of hPDL-MSCs stimulated with LPS (1 µg/ml) for 24 h. **(b)** Effect of resveratrol on the viability of SAOS-2 cells stimulated with LPS (1 µg/ml) for 24 h. **(c)** Effect of resveratrol on the viability of hPDL-MSCs stimulated with LPS (1 µg/ml) for 24 h. **(d)** Data are expressed as mean ± standard deviation (*n* = 9).Statistical analysis was applied by ANOVA test and Dunnett's multiple comparison post-test (*p < 0.05, ***p < 0.001), CT, control, cells plus LPS; VH, DMSO vehicle plus LPS; ISO, isodrimeninol; RESV, resveratrol; LPS, lipopolysaccharide.

Cell viability of isodrimeninol and resveratrol was assessed by MTS assays in Saos-2 cells (Figure 1) and hPDL-MSCs stimulated with 1 µg/ml LPS for 24 h. After exposing both cell cultures to concentrations of 0 (vehicle and control), 6.25, 12.5, 25, and 50 µg/ml of isodrimeninol, cell viability was found to be in the acceptable range (>90%) at the concentrations evaluated in Saos-2 cells (Supplementary Figure 1a) and hPDL-MSCs (Supplementary Figure 1b). However, when stimulated with 1 µg/ml of LPS, the concentrations of 25 µg/ml (*p* < 0. 05) and 50 µg/ml (*p* < 0.001) were toxic to Saos-2 cells (Figure 2a) and hPDL-MSCs (Figure 2b) in 24 h. Regarding the comparison of resveratrol toxicity on Saos-2 cells and hPDL-MSCs, the concentrations used of 0 (vehicle and control), 5.71, 11.41 and 22.82 µg/ml did not affect cell viability in Saos-2 cells (Supplementary Figure 1c) and hPDL-MSCs (Supplementary Figure 1d); however, when stimulated with 1 µg/ml of LPS for 24 h, the 22.82 µg/ml concentration was toxic for both cell models, thus considerably decreasing cell viability (*p* < 0. 001 for Saos-2 cells and hPDL-MSCs (Figure 2c,d, respectively). In this work, concentrations of 6.25 and 12.5 µg/ml of isodrimeninol and 5.71 and 11.41 µg/ml of resveratrol were selected in the subsequent experiments, ensuring a viability close to 100%.

### 3.2 Effect of isodrimeninol from *Drimys winteri* and resveratrol on the expression of pro-inflammatory cytokines in Saos-2 cells and hPDL-MSCs

**Figure 3:**
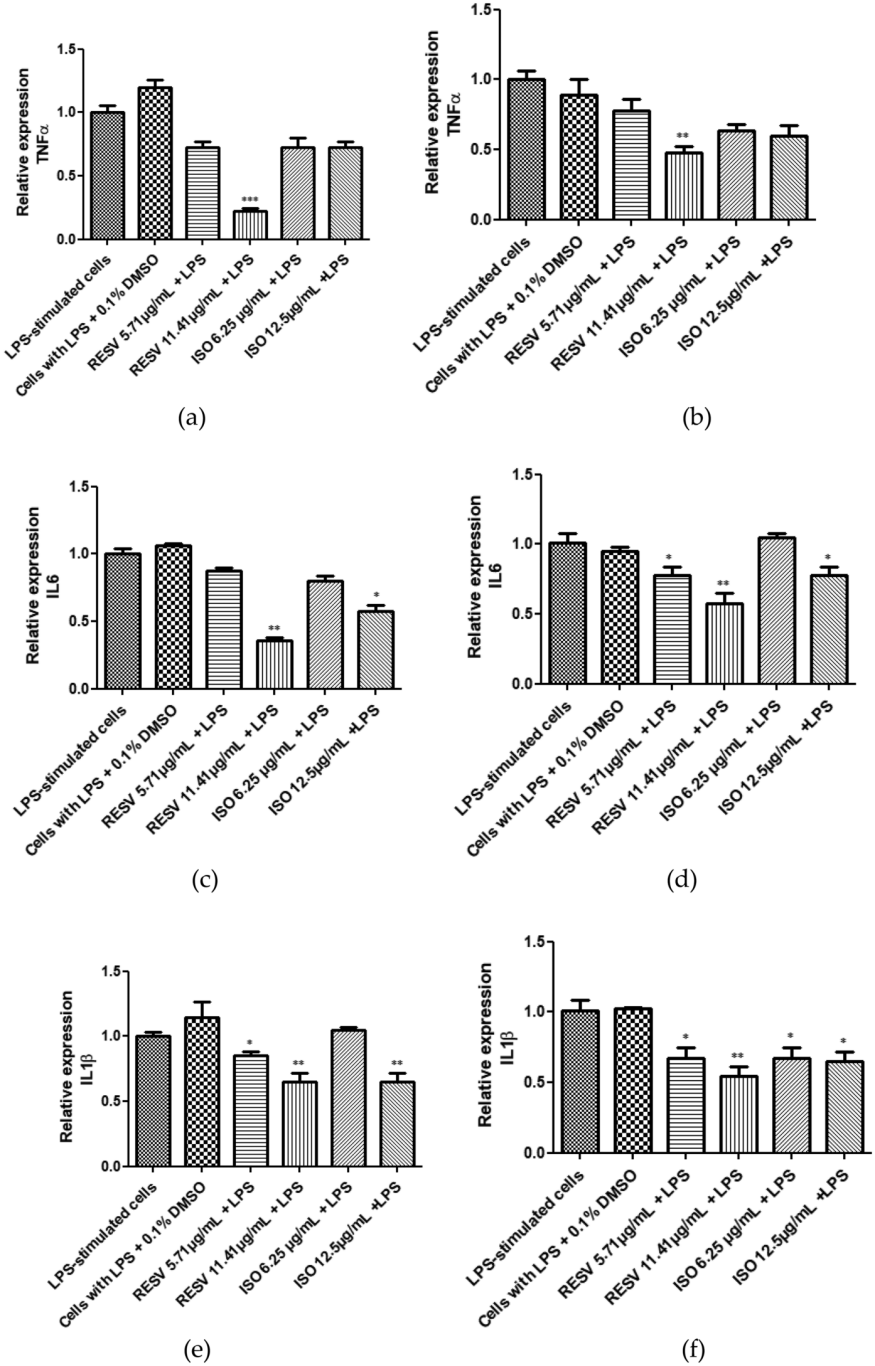
Effect of isodrimeninol from *Drimys winteri* and resveratrol on IL-6, TNF-α, and IL-1β gene expression in saos-2 cells **(a,c,e)** and hPDL-MSCs **(b,d,f)** stimulated with 1 µg/ml LPS and treated with Dw isodrimeninol at concentrations of 6.25 and 12.5 µg/ml and resveratrol at concentrations of 5.71 and 11.41 µg/ml for 24 h. q-PCR was normalized against RPL-27. Bars represent the mean expression for each group ± standard deviation. Statistical analysis was performed using ANOVA and Dunnett's multiple comparisons post-test (*p < 0.05, **p < 0.001 y ***p < 0.0001). CT, control, LPS-stimulated cells; VH, cells stimulated with LPS + 0.1% DMSO; TNF-α, tumor necrosis factor; IL-6, interleukin 6; IL-1β, interleukin 1 beta; ISO, isodrimeninol; RESV, resveratrol.

The effect of *Dw* isodrimeninol and resveratrol as a positive control on gene expression of pro-inflammatory cytokines such as IL-6, TNF-α, and IL-1β in Saos-2 cells and hPDL-MSCs stimulated with 1 µg/ml of LPS for 24 h was evaluated by qRT-PCR (Figure 3). The results showed that isodrimeninol (12.5 µg/ml) significantly decreased IL-6 expression (*p* < 0.0001) in Saos-2 cells compared to the control (cells stimulated with 1ug/ml LPS) (Figure 3c) and hPDL-MSCs (Figure 3d), and the same occurred with IL-1β expression in SAOS-2 cells (*p* < 0.0001) (Figure 3e). In hPDL-MSCs (Figure 3f), both concentrations (6.25 and 12.5 µg/ml) of isodrimeninol significantly decreased IL-1β expression (*p* < 0.0001). However, TNF-α showed no significant difference in its expression when treated with isodrimeninol (6.25 and 12.5 µg/ml) compared to the control (cells stimulated with 1 µg/ml LPS) in both cell cultures (Figures 3a,b). Regarding the resveratrol treatment, it significantly decreased IL-6, TNF-α, and IL-1β expression at the 11.41 µg/ml concentration in Saos-2 cells (Figures 3a,c,e) and hPDL-MSCs (Figures 3b,d,f) stimulated with LPS compared to the control. In addition, resveratrol treatment at the 5.71 µg/ml concentration significantly decreased IL-6 expression in hPDL-MSCs (Figure 3d) and IL-1β in both cell cultures (Figures 3e,f).

### 3.3 Expression of miRNAs in Saos-2 cells and hPDL-MSCs

**Figure 4:**
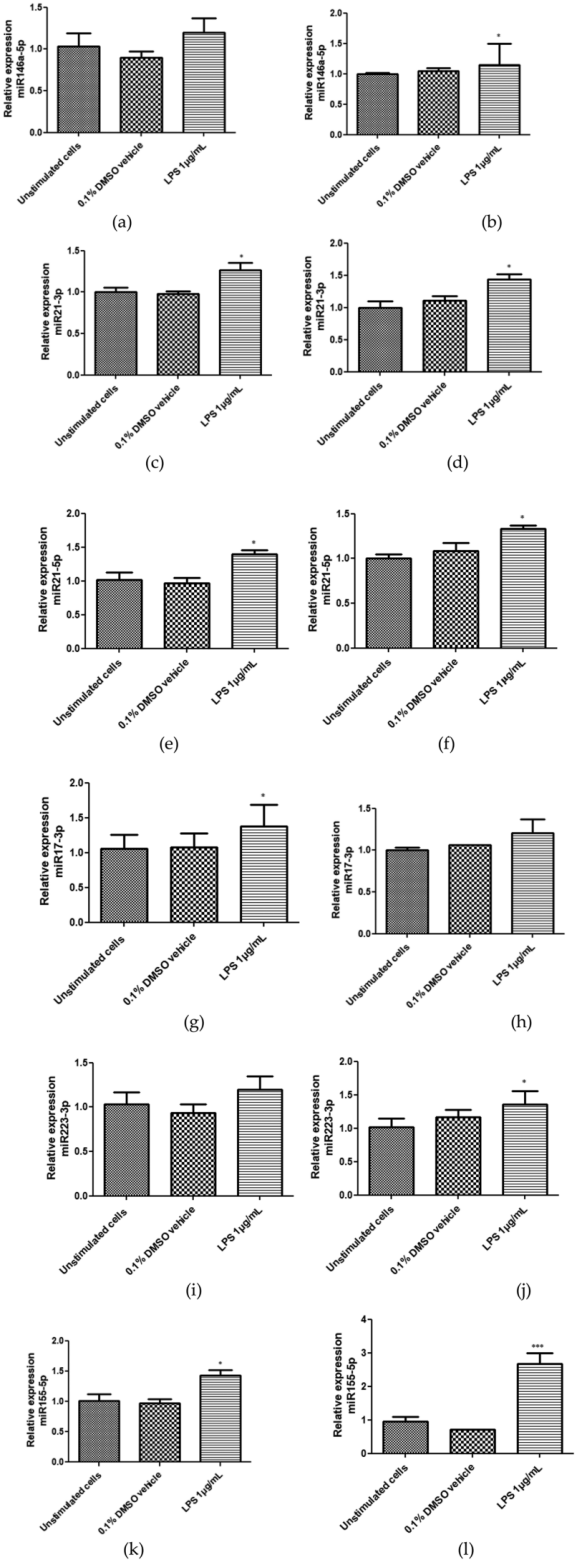
Relative expression of hsa-miR-146a-5p, hsa-miR-21-3p, hsa-miR-21-5p, hsa-miR-17-3p, hsa-miR-223-3p, and hsa-miR-155-5p in saos-2 cells **(a,c,e,g,i,k)** and hPDL-MSCs **(b,d,f,h,j,l)** stimulated with 1 µg/ml LPS for 24 h. miRNA expression was evaluated by q-PCR. To normalize expression, hsa-miR-191-5p was used as an endogenous control. The bars represent the mean expression for each group ± standard deviation. Statistical analysis was applied by ANOVA test and Dunnett's multiple comparisons post-test (*p < 0.05). CT, control, unstimulated cells with LPS; VH, 0.1% DMSO vehicle; LPS, lipopolysaccharide.

Expression of hsa-miR-17-3p, hsa-miR-21-3p, hsa-miR-21-5p, hsa-miR-146a-5p, hsa-miR-155-5p, and hsa-miR-223-3p was evaluated in Saos-2 cells and hPDL-MSCs unstimulated and stimulated with LPS (1 µg/ml) for 24 h (Figures 4a-l). Hsa-miR-146a-5p and hsa-miR-223-3p showed a significant increase in their expression in hPDL-MSCs stimulated with 1 µg/ml LPS compared to the control (Figures 4b,j). The microRNAs hsa-miR-21-3p, hsa-miR-21-5p, and hsa-miR-155-5p significantly increased their expression in LPS-stimulated Saos-2 cells (Figures 4c,e,k) and hPDL-MSCs (Figures 4d,f,l) compared to the control cells. In addition, hsa-miR-17-3p showed a significant increase in its expression in LPS-stimulated Saos-2 cells (Figure 4g) compared to the control cells.

### 3.4 Effect of isodrimeninol from *Drimys winteri* and resveratrol on miRNA expression in Saos-2 cells and hPDL-MSCs

**Figure 5:**
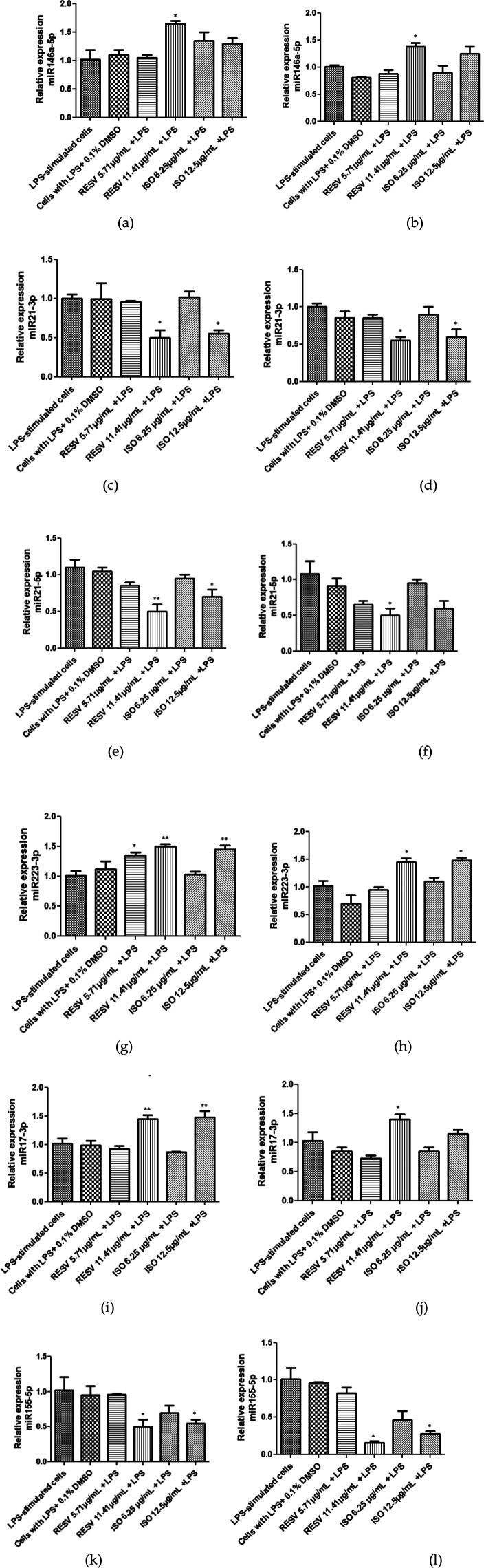
Effect of isodrimeninol from *Drimys winteri* and resveratrol on the gene expression of hsa-miR-146a-5p, hsa-miR-21-3p, hsa-miR-21-5p, hsa-miR-223-3p, hsa-miR-17-3p, and hsa-miR-155-5p in saos-2 cells **(a,c,e,g,i,k)** and hPDL-MSCs **(b,d,f,h,j,l)** stimulated with 1 µg/ml LPS for 24 h. The gene expression of miRNAs was assessed by q-PCR. To normalize expression, hsa-miR-191-5p was used as an endogenous control. The bars represent the mean expression for each group ± standard deviation. Statistical analysis was applied by ANOVA test and Dunnett's multiple comparisons post-test (*p < 0.05, **p < 0.001). CT, control, LPS-stimulated cells; VH, cells stimulated with LPS + 0.1% DMSO; LPS, lipopolysaccharide; RESV, resveratrol; ISO, isodrimeninol.

Figure 5 show the expression levels of hsa-miR-146a-5p, hsa-miR-21-3p, hsa-miR-21-5p, hsa-miR-17-3p, hsa-miR-223-3p, and hsa-miR-155-5p in Saos-2 cells and hPDL-MSCs stimulated with LPS (1 µg/ml) and treated with concentrations of 0 (control and vehicle), 6.25, and 12.5 μg/ml isodrimeninol for 24 h and with concentrations of 0 (control and vehicle), 5.71 and 11.41 µg/ml of resveratrol. Hsa-miR-146a-5p showed no significant difference in expression when treated with isodrimeninol (6.25 and 12.5 μg/ml) in LPS-stimulated Saos-2 cells (Figure 5a) and hPDL-MSCs (Figure 5b). In contrast, resveratrol (11.41 µg/ml) positively regulated the expression of this miRNA in both cell cultures. Furthermore, resveratrol, in both cell cultures, at one or both of the tested concentrations, led to a significant increase in the expression of hsa-miR-223-3p (Figures 5g,h), hsa-miR-17-3p (Figures 5i,j), and hsa-miR-155-5p (Figures 5k,l), and conversely led to a decrease in the expression of hsa-miR-21-3p (Figures 5c,d) and hsa-miR-21-5p (Figures 5e,f). Isodrimeninol (12.5 µg/ml) negatively regulated the expression of hsa-miR-21-3p, hsa-miR-21-5p and hsa-miR-155-5p in Saos-2 cells (Figures 5c,e,k) and hsa-miR-21-3p (Figure 5d) and hsa-miR-155-5p (Figure 5l) in LPS-stimulated hPDL-MSCs compared to untreated LPS-stimulated cells. In contradiction to previous results, isodrimeninol (12.5 µg/ml) significantly increased hsa-miR-223-3p expression in Saos-2 cells (Figure 5g) and hPDL-MSCs (Figure 5h) and positively regulated hsa-miR-17-3p expression in LPS-stimulated Saos-2 cells (Figure 5i).

## 4 Discussion

MiRNAs play a pivotal role in the inflammatory processes underlying periodontitis by modulating gene expression and immune responses (12). Understanding how miRNAs control the immune system is vital for developing early detection and treatment methods for inflammatory conditions like periodontitis. Specific miRNAs, such as hsa-miR-17, miR-155, and miR-146a, show alte4red expression in periodontal disease (32), coinciding with our results. One of the initial events of the inflammatory response to microbial challenge in the gingival sulcus is neutrophil migration to inflammation sites, which is regulated by the expression of selectin E and intercellular adhesion molecule-1 (ICAM-1), targets of miR-17-3p, respectively. It has been observed that specific antagonists of miR-17-3p increase neutrophil adhesion to endothelial cells, whereas mimics of this miRNA elicit the opposite response (11, 33). Additionally, a recent study demonstrated that LPS causes positive regulation of miR-17-3p in mice and human umbilical vein endothelial cells (HUVEC) and that overexpression of miR-17-3p suppresses LPS-induced NF-κB activation (34). miR-155 and miR-146a are also involved in the regulation of the NF-κB signaling pathway; for example, miR-155, which is positively regulated by TNF-α in HUVEC, suppresses the NF-κB signaling pathway in atherosclerosis by targeting transcription factor p65 and decreasing monocyte adhesion to the endothelium (35). It has also been reported that miR-155 negatively regulates inflammatory cytokine production and slows the progression of atherosclerosis because it controls the inflammatory response by repressing the mitogen-activated protein kinase 10 (MAP3K10) pathway (36). This stands in contrast to other researchers (37) who found that miR-155 directly suppressed the expression of B-cell lymphoma 6 (BCL6) protein, a transcription factor that attenuates pro-inflammatory NF-κB signaling.

On the other hand, several researchers have shown in their study an increased expression of miR-146a in patients with generalized aggressive periodontitis compared to healthy subjects, and overexpression of this miRNA was accompanied by reduced levels of pro-inflammatory cytokines (38), coinciding with the results yielded by another study into chronic periodontitis (26). It has also been shown that after stimulation of THP-1 cells with LPS and pro-inflammatory cytokines, miR-146a expression was induced through an NF-κB-dependent pathway, resulting in the negative regulation of adaptor proteins involved in inflammatory responses such as TNF receptor-associated factor 6 (TRAF6) and interleukin-1 receptor-associated kinase 1 (IRAK1); consequently, miR-146a participates as a negative regulator of inflammation (39).

In the case of hsa-miR-21, previous studies have reported that miR-21-3p is among the most important biomarkers in periodontal disease, which is related to the MAPK tumor signaling pathway, T lymphocyte receptors, adhesion molecules, and others (40). Coinciding with our results, it has been shown that miR-21 has a higher expression in macrophages stimulated with LPS and that its deficit increased the production of pro-inflammatory cytokines and promoted the activation of NF-κB and vice versa, displaying its anti-inflammatory function (9). In addition, miR-21 modulates the inflammatory response by differentially regulating the expression of IL-1β and IL-10 (41–44), and they also reported an increased expression of hsa-miR-21 in patients with chronic periodontitis compared to healthy individuals. In contrast, other authors found decreased expression of serum miR-21-3p levels in periodontitis in their microarray analysis; however, real-time PCR analysis indicated increased expression of this miRNA (37). Furthermore, in another recent study, miR-21-3p expression was also reduced, but in patients with peri-implantitis compared to peri-implant mucositis sites (45).

Regarding miR-223-3p, other researchers found a reduced expression of this miRNA in salivary exosomes in periodontitis compared with healthy controls, which contradicts our results. They further found that the pyrin 3 (NLRP3) domain of the (NOD)-like receptor (NLR), which is a key mediator in the production of IL-1 family cytokines in periodontitis, was targeted by miR-223-3p because when they eliminated miR-223-3p expression in THP-1-derived macrophages, the expression of NLRP3 and the inflammatory mediators IL-1β and IL-6 increased (46, 47). Consistent with these results, it has been reported that miR-223-3p expression was lower in saliva samples from patients with periodontitis (48). However, other authors agreeing with us showed that miR-223 expression was significantly increased in inflamed gingival tissues and was positively correlated with the clinical parameters of periodontitis (49). Moreover, miR-223 has been reported to be significantly overexpressed in both serum and gingival crevicular fluid of patients with periodontitis (50). All these contradictory results may be due to variability in study design and different sample and cell types.

Given the limitations and adverse effects observed in traditional periodontitis treatment, such as bacterial resistance to antimicrobial agents (51), it is essential to study natural compounds that can hinder the development of periodontopathogenic bacteria, alter the host inflammatory response, suppress local periodontal inflammation, and specifically lessen NF-κB activation. With this context in mind, our study investigated the impact of isodrimeninol derived from *Dw* on the expression of pro-inflammatory cytokines and miRNAs that are deregulated in periodontitis, focusing on inflammatory pathways in Saos-2 cells and hPDL-MSCs stimulated with LPS from *P. gingivalis*. For this, we used resveratrol (3, 4′, 5-trihydroxystilbene), a native compound from the polyphenolic group of stilbenes, as a positive control since it has been shown to possess antibacterial, anti-adherence, and antiprotease properties against *P. gingivalis*, decreasing this pathogen-mediated activation of the NF-κB signaling pathway (52). Regarding isodrimeninol, a compound we evaluated, it was isolated from *Dw* (53). *Dw* has been reported as having antinociceptive (54), insecticidal (55), antifungal (56), and anti-inflammatory (21) activity. Previous studies (21) have shown in an *in vitro* atherosclerosis model that total *Dw* extract, as well as drimenol, isodrimeninol, and polygodial at 10 μg/ml, inhibit the adhesion of THP1 cells such as blood monocytes to TNF-α-stimulated human umbilical vein endothelial cells (HUVEC) and reduced TNF-α-induced overexpression in HUVEC of vascular cell adhesion molecule-1 (VCAM-1), which is key in the regulation of vascular inflammation, where monocyte adhesion and their transmigration into the intima starts a cascade of inflammatory reactions (57, 58). *Dw* and isodrimeninol have also been reported to induce anti-atherosclerotic effects by inhibiting foam cell formation in macrophage M1 and promoting anti-inflammatory responses, as they improved the expression of anti-inflammatory cytokine IL-10 and significantly reduced IL-1β expression (22). Based on this evidence, we confirmed the anti-inflammatory effect of isodrimeninol, suggesting that it could be used as a complementary treatment in periodontal therapy, although further research is needed to support our results. However, resveratrol treatment exhibited a greater anti-inflammatory capacity.

Isodrimeninol treatment of LPS-stimulated Saos-2 cells and hPDL-MSCs had no significant effect on TNF-α expression (Supplementary results), consistent with previous studies (22). In the case of miRNAs, treatment with isodrimeninol at a concentration of 12.5 µg/ml caused a significant decrease in the expression of miR-21-3p in both cell cultures and of miR-21-5p in Saos-2 cells. It should be noted that miR-21 has an anti-inflammatory effect since its deficit increases the production of pro-inflammatory cytokines and the activation of NF-κB (9). This result contradicts the anti-inflammatory effect of isodrimeninol, so further studies should be conducted to verify and examine possible reasons for this effect, similar to that caused by resveratrol, although we know that the mechanisms of miRNA regulation are complex. Contradictorily, at the 12.5 µg/ml concentration, isodrimeninol showed a significant increase of miR-17-3p expression in Saos-2 cells but caused a significant reduction of miR-155-5p in both cell cultures while increasing miR-223-3p expression in both Saos-2 cells and hPDL-MSCs. Since the overexpression of this miRNA has been shown to inhibit the activation of this inflammation signaling pathway (59), the increased expression of miR-17-3p, further elicited by isodrimeninol treatment, contributes to inhibiting NF-κB. This suggests that the anti-inflammatory effect of isodrimeninol and resveratrol may be mediated by negative regulation of the NF-κB pathway and by negative regulation of the expression of miR-155. It also indicates that miR-155 could be a useful agent in treating inflammatory diseases since miR-155 is known for its pro-inflammatory role in the LPS-stimulated immune response (60). Regarding the expression of miR-223-3p, isodrimeninol showed a significant increase in the expression of this miRNA at the 12.5 µg/ml concentration in both cultures, considering that the increased expression of miR-223-3p decreases the expression of NLRP3, which is a key mediator in the production of IL-1 family cytokines in periodontitis (46, 47). According to those results and our own, we can suggest that isodrimeninol exerts its anti-inflammatory effects by modulating the NF-κB signaling pathway. NF-κB is a key transcription factor that regulates the expression of pro-inflammatory cytokines (9) and adhesion molecules (22). By inhibiting the phosphorylation and degradation of IκBα, an inhibitor of NF-κB, isodrimeninol likely prevents the nuclear translocation and activation of NF-κB (22). This, in turn, could suppress the production of inflammatory mediators such as TNF-α and IL-1β, as observed in our study. Moreover, long non-coding RNAs (lncRNAs) like MALAT1 have been found to influence the miR-146a/NF-κB signaling pathway, thereby impacting inflammatory responses (61). However, the exact molecular target of isodrimeninol remains to be elucidated. It is possible that this compound interacts with upstream regulators of NF-κB, such as IKKβ or NIK. Further mechanistic studies are warranted to fully understand how isodrimeninol modulates inflammatory responses at the molecular level, recognizing this as a limitation of this study.

Isodrimeninol and other drimane sesquiterpenoids derived from *Dw* hold promise as natural scaffolds for the development of anti-inflammatory drugs. However, the current findings are based on *in vitro* studies, which have inherent limitations. *In vitro* cell culture models, such as Saos-2 and hPDL-MSCs, lack the complex three-dimensional architecture, cell-cell interactions, vascularization, and biomechanical forces present *in vivo*, which can significantly influence cell behavior and drug responses (62). Moreover, these models struggle to capture long-term effects and systemic interactions between different cell types and organs that occur in the body. Therefore, further research utilizing *in vivo* disease models and clinical trials is necessary to validate the efficacy and safety of isodrimeninol for specific indications, such as periodontal disease. Investigating the pharmacokinetics, bioavailability, and potential toxicity of isodrimeninol will also be crucial for clinical translation. Additionally, quantifying pro-inflammatory cytokines at the protein level would be important, particularly due to the diverse implications of post-translational modifications that could enhance the preliminary results obtained in this study. Furthermore, incorporating other types of periodontal cells, such as epithelial cells, connective tissue cells (fibroblasts), or monocytic/macrophagic cells, would provide a more comprehensive and accurate evaluation. Alternatively, using another cell line with characteristics similar to osteoblasts, distinct from Saos-2, could also be beneficial.

## 5 Conclusions

The findings of this study underscore the potential of isodrimeninol as a novel therapeutic agent for modulating miRNA expression associated with periodontitis. These results position isodrimeninol as a promising candidate for complementary periodontal therapy, particularly in mitigating inflammation-driven tissue destruction. However, further research utilizing *in vivo* models is crucial to validate these findings and evaluate their clinical applicability.

## Data Availability

The raw data supporting the conclusions of this article will be made available by the authors, without undue reservation.
