# Supplementary material for: MicroRNAs modulation by isodrimeninol from Drimys winteri in periodontitis-associated cellular models: preliminary results
Source: Front Oral Health. 2025 May 21;6:1489823. doi: 10.3389/froh.2025.1489823 (PMC12133741; doi:10.3389/froh.2025.1489823)
Supplement: Supplementary file 1 [file Datasheet1.pdf]

Supplementary Material

1 Supplementary Figures

Effect of LPS from Porphyromonas gingivalis on the viability of Saos-2 cells and hPDL-MSCs.

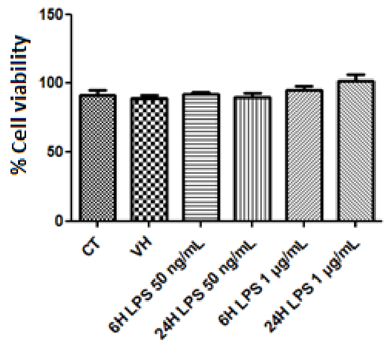

(a)

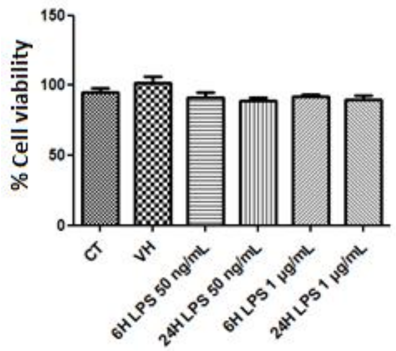

(b)

Supplementary Figure 1. Effect of *Porphyromonas gingivalis* LPS (50 ng/mL and 1 µg/mL) on the viability of Saos-2 cells and hPDL-MSCs after 6 and 24 hours, assessed via MTS assay. Statistical analysis (ANOVA and Dunnett's post-test) compared control (CT), vehicle (VH), and LPS-treated groups, with data presented as mean ± SD (n = 9).

Development of the inflammatory model in Saos-2 cells and hPDL-MSCs.

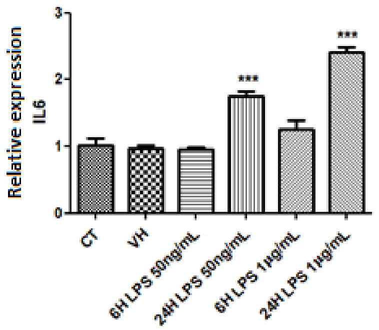

(a)

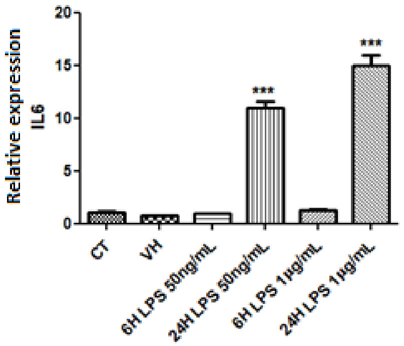

(b)

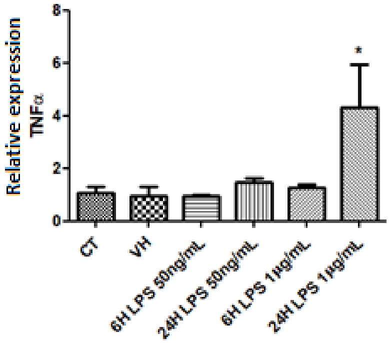

(c)

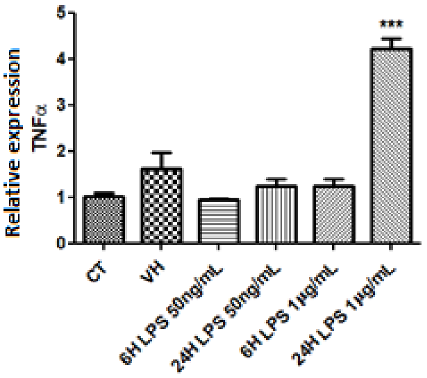

(d)

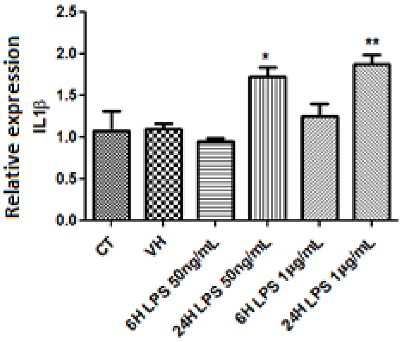

(e)

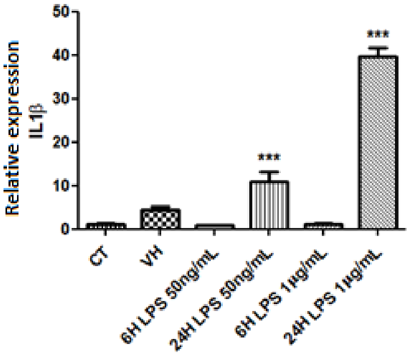

(f)

Supplementary Figure 2. Expression of IL-6, TNF-α, and IL-1β in Saos-2 cells and hPDL-MSCs after stimulation with 50 ng/mL and 1 µg/mL of LPS for 6 and 24 hours. Data are normalized to RPL27 and analyzed using ANOVA and Dunnett's post-test. Significant differences are indicated by asterisks (*p<0.05, **p<0.001, ***p<0.0001).
