# Supplementary material for: MicroRNAs modulation by isodrimeninol from Drimys winteri in periodontitis-associated cellular models: preliminary results
Source: Front Oral Health. 2025 May 21;6:1489823. doi: 10.3389/froh.2025.1489823 (PMC12133741; doi:10.3389/froh.2025.1489823)
Supplement: Supplementary file 2 [file Datasheet2.pdf]

1 Supplementary Data

1.1. Standardization of LPS dose as a stimulus for the inflammation model: Saos-2 cells and hPDL-MSCs were cultured under the conditions described above. Once a 70-80% confluence percentage was reached, the cells were seeded in a 96-well plate at a cell concentration of $1x10^5$ cells/well to perform cell viability assays. After 24 hours of incubation, the culture medium was replaced with a medium supplemented with LPS of P. gingivalis from Sigma, Aldrich (SMBOO610) at concentrations of 50 ng/mL and 1 µg/mL, left to incubate for 6 and 24 hours. After that time, the culture medium was removed and replaced with 20 µL of CellTiter 96® AQueous One Solution reagent (MTS; Promega, Madison, WI, USA) in each well, followed by incubation for 4 hours at 37°C. Viability assays were performed according to the manufacturer's protocols. Absorbance was determined using a microplate reader (NanoQuant, Infinite® M200PRO-Tecan, Redwood, CA, USA) at 490 nm. The evaluation included controls: cells only with culture medium and cells with culture medium and the solvent or vehicle used to prepare the LPS, which was 0.1% dimethyl sulfoxide (DMSO) (Invitrogen). The assays were performed in technical and biological triplicates, and the results were expressed as the percentage of viability compared to the vehicle. Parallel seeding was performed in 24-well plates at $1x10^5$ cells/well concentrations. Cells were treated with the abovementioned conditions (50 ng/mL and 1 µg/mL LPS being left to incubate for 6 and 24 hours). Gene expression of the pro-inflammatory cytokines IL-6, TNF-α, and IL-1β was assessed to test for inflammation.

1.2. Effect of treatment with different doses of Drimys winteri isodrimeninol and resveratrol on cell viability in Saos-2 cells and hPDL-MSCs. For the *in vitro* viability assays of Saos-2 cells and hPDL-MSCs stimulated or not with LPS (1 µg/mL), the MTS cell proliferation assay was used to determine the cytotoxic effect of resveratrol and isodrimeninol. Isodrimeninol was previously extracted from Dw bark, purified by silica gel column chromatography, and characterized by gas chromatography, mass detector, and nuclear magnetic resonance spectroscopy, presenting a purity of 90% and molar mass of 236 g/mol. Finally, 0.25 mg of isodrimeninol was dissolved in 50 µL of DMSO to obtain a 50 mg/mL stock solution, and the solutions were kept at -20°C until use. The solutions were filtered through a sterile 0.22 µm filter and used to prepare the desired treatments. Saos-2 cells and hPDL-MSCs were seeded in 96-well plates ($1x10^5$ cells/well) and stimulated with 1 µg/mL LPS for 24 hours. They were then exposed to different concentrations of isodrimeninol (6.25, 12.5, 25, 50 µg/mL) and resveratrol (5.71, 11.41, 22.82 µg/mL), obtained from Sigma Aldrich (Cat No. R5010) and used as a positive control, and maintained in culture for 24 hours. After the treatment, the culture medium was removed and replaced by 20 µL of MTS reagent in each well, followed by incubation for 4 h at 37ºC. Absorbance was determined using a microplate reader (NanoQuant, Infinite® M200PRO–Tecan, Redwood, CA, USA) at 490 nm. Isodrimeninol and resveratrol were dissolved in DMSO. The final concentration of the solvent in the culture medium did not exceed 0.1%. The evaluation included controls: cells only with culture medium and cells with culture medium and the solvent or vehicle used to prepare the treatments (0.1% DMSO). The assays were performed in technical and biological triplicates, and the results were expressed as the percentage of viability compared to the vehicle.
